# A Three-Component Microbial Consortium from Deep-Sea Salt-Saturated Anoxic Lake Thetis Links Anaerobic Glycine Betaine Degradation with Methanogenesis

**DOI:** 10.3390/microorganisms3030500

**Published:** 2015-09-09

**Authors:** Violetta La Cono, Erika Arcadi, Gina La Spada, Davide Barreca, Giuseppina Laganà, Ersilia Bellocco, Maurizio Catalfamo, Francesco Smedile, Enzo Messina, Laura Giuliano, Michail M. Yakimov

**Affiliations:** 1 Institute for Coastal Marine Environment, CNR, Spianata S. Raineri 86, Messina 98122, Italy; E-Mails: violetta.lacono@iamc.cnr.it (V.L.C.); eren@tiscali.it (E.A.); gina.laspada@iamc.cnr.it (G.L.S.); maurizio.catalfamo@iamc.cnr.it (M.C.); francesco.smedile@iamc.cnr.it (F.S.); enzo.messina@iamc.cnr.it (E.M.); laura.giuliano@iamc.cnr.it (L.G.); 2 Department of Organic and Biological Chemistry, University of Messina, Salita Sperone 31, Villaggio S. Agata, Messina 98166, Italy; E-Mails: dbarreca@unime.it (D.B.); giuseppina.lagana@unime.it (G.L.); ersiliasanta.belocco@unime.it (E.B.); 3 Mediterranean Science Commission (CIESM), 16 bd de Suisse, MC 98000, Monaco

**Keywords:** deep-sea hypersaline anoxic lakes, redoxicline, glycine betaine degradation, methylotrophic halophiles, methanogenesis

## Abstract

Microbial communities inhabiting the deep-sea salt-saturated anoxic lakes of the Eastern Mediterranean operate under harsh physical-chemical conditions that are incompatible with the lifestyle of common marine microorganisms. Here, we investigated a stable three-component microbial consortium obtained from the brine of the recently discovered deep-sea salt-saturated Lake Thetis*.* The trophic network of this consortium, established at salinities up to 240, relies on fermentative decomposition of common osmoprotectant glycine betaine (GB). Similarly to known extreme halophilic anaerobic GB-degrading enrichments, the initial step of GB degradation starts with its reductive cleavage to trimethylamine and acetate, carried out by the fermenting member of the Thetis enrichment, *Halobacteroides lacunaris* TB21. In contrast to acetate, which cannot be easily oxidized in salt-saturated anoxic environments, trimethylamine represents an advantageous C_1_-substrate for methylotrophic methanogenic member of the Thetis enrichment, *Methanohalophilus* sp. TA21. This second member of the consortium likely produces hydrogen via methylotrophic modification of reductive acetyl-CoA pathway because the initial anaerobic GB cleavage reaction requires the consumption of reducing equivalents. Ecophysiological role of the third member of the Thetis consortium, *Halanaerobium* sp. TB24, which lacks the capability of either GB or trimethylamine degradation, remains yet to be elucidated. As it is true for cultivated members of family *Halanaerobiaceae*, the isolate TB24 can obtain energy primarily by fermenting simple sugars and producing hydrogen as one of the end products. Hence, by consuming of TB21 and TA21 metabolites, *Halanaerobium* sp. TB24 can be an additional provider of reducing equivalents required for reductive degradation of GB. Description of the Thetis GB-degrading consortium indicated that anaerobic degradation of osmoregulatory molecules may play important role in the overall turnover of organic carbon in anoxic hypersaline biotopes.

## 1. Introduction

Since the discovery on the Mediterranean seabed of two deep-sea hypersaline anoxic lakes (DHALs) [1,2,3], yet more are being discovered and currently there are eight such hydrological formations known [4,5,6]. The surface of these brine lakes lies 3.0–3.5 km below sea level and the salinity of the brines is five to thirteen times higher than that of seawater. Nascency of these peculiar hydrological formations is related to dissolution of the ancient salt deposits (*ca.* 5–6 million years old), called Messinian evaporites. Our studies of DHALs have shown that in spite of being so harsh, the brine and, especially, interface of these lakes host the active and strongly stratified microbial communities [5,6,7,8,9]. Overwhelming majority of DHAL autochthonous prokaryotes currently resist cultivation and very few of the isolates and enrichments were obtained thus far from deep-sea anoxic lakes [9,10,11,12,13,14].

The main factors that determine whether a certain type of microorganisms can live at extreme salinity are the amount of energy generated during dissimilatory reactions and the mode of maintaining of osmotically-balanced and functional cytoplasm [15]. The most halophilic and halotolerant aerobic microorganisms meet the osmolarity challenge by synthesis and maintaining high intracellular concentrations of osmoprotectants or compatible solutes that act as osmolytes. This type of haloadaptation, known as “salt out” strategy, requires high energetic costs and, thus, limits the number of organisms that can thrive in dark, anoxic, salt-saturated, and energy-impoverished environments where photosynthesis, aerobic respiration, and other highly exergonic processes cannot be operative. Nonetheless, there are many evidences in existence of complex microbial communities thriving under such polyextremophilic conditions [16]. Only few groups of exteremely halophilic strict anaerobes are cultured to date in laboratories. They mainly belong to methylotrophic methanogenic euryarchaea of the order Methanosarcinales and to metabolically versatile fermenting bacteria of the order Halanaerobiales*.* Despite belonging to different prokaryotic domains, these organisms possess a striking similarity of haloadaptation and energy conservation mechanism, which is considered as one of the simplest and ancient types of metabolism [17]. Instead of synthesis of the organic osmolytes, these prokaryotes accumulate high intracellular concentrations of sodium (up to 1 M) and potassium ions (up to 4–5 M) to maintain the intracellular turgor and to withstand against osmotic stress of hypersaline environment (“salt inside” strategy). To produce reduced equivalents at near saturation salinities some of these anaerobic extreme halophiles can rely upon disproportionation of methylated compounds [15,16,18]. These reactions occur via cognate methylotrophic modifications of reductive acetyl-CoA pathway, acetogenic in bacteria [19], and methanogenic in archaea [20].

In addition to sharing physiological similarities, some of these strict anaerobic extreme halophiles are syntrophically linked to each other, thus essentially constituting a single functional entity of microbial communities. Methylated compounds, such as trimethylamines (TMA), are among the fundamental catabolic substrates fueling and sustaining this trophic interaction [9,15,16,21,22]. TMA is often found in saline systems, where it originated from degradation of glycine betaine (GB) or other organic osmoprotectants (compatible solutes) produced by a variety of moderate halophiles possessing “salt out” adaptation. These organisms typically thrive in the superficial and less-salted aerobic layers [21,22]. There are very few studies describing GB-based trophic networks in salt-saturated environments [9,18,23,24]. Apparently, in oxygen-depleted ecosystems under even moderate salinity (120–150) neither sulfate reducers nor methanogens can utilize GB directly. It requires activity of halophilic fermenting members of community, reductively degrading GB to trimethylamine and acetate [21,22]. A quarter of a century ago two extremely halophilic organisms, involved in trophic syntrophy, were isolated from hypersaline anoxic sediments of Arabat spit (East Crimea, Ukraine) [24]. This simple consortium consisted of the homoacetogenic bacterium *Acetohalobium arabaticum* and the methylotrophic, H_2_-generating, methanogenic archaeon *Methanohalobium evestigatum.* The homoacetogen degraded GB and produced trimethylamine as one of the intermediates, which was further consumed by a methanogenic member of the consortium.

Recently [9] we proposed that evolution of both acetate and methane in salt-saturated brines of the deep-sea hypersaline anoxic Lake Medee is at least partially based on a C_1_-syntrophic network similar to that described above. Here we present an evidence that this type of syntrophy can also take place in other Mediterranean DHALs, where both methano- and acetogenesis are well documented [7,8,25]. Using a previously-designed cultivation approach [9] we were able to obtain a stable, GB-degrading, strict anaerobic, three-component microbial consortium from deep-sea hypersaline Lake Thetis(3500 m depth), which links GB degradation with methanogenesis. To study the metabolic preferences of the GB-degrading consortium and the effect of *in situ* pressure conditions on its members, we isolated them and cultivated separately.

## 2. Materials and Methods

### 2.1. Enrichment and Isolation

Brine samples (salinity, 340) of the anoxic Lake Thetis (34.6698° N and 22.1455° E) were collected during MICRODEEP12 cruise (September 2012) on board of R/V *Urania*. For cultivation, brine was supplemented with yeast extract (20 mg·L^−1^), selenite/tungstate (10 mg·L^−1^), trace elements, and vitamin solution (1 mL·L^−1^ each) (see DSMZ medium 141 for detailed reference). The medium was previously adjusted to pH 7.0 with 1.0 M NaOH, boiled under N_2_-CO_2_ atmosphere (80:20 v/v), dispensed into 120-mL serum bottles under N_2_:CO_2_ (80:20 v/v) and autoclaved. After sterilization, 0.5 g·L^−1^ filter sterilized Na_2_S and 10 mM GB were added. The medium was anaerobically inoculated with the brine sample to achieve the stepwise dilutions (final salinities 300, 260, 240, 240, and 200). Cultures were incubated at *in situ* temperature in the dark for two months. CARD-FISH analysis and CH_4_ production were performed as detailed below. The saltiest CH_4_-producing Thetis enrichment (salinity, 240) was transferred into fresh medium supplemented with 10 mM of GB and used for further isolation and phylogenetic analysis. Minimal medium (salinity, 160) supplemented with 20 mM dimethylamine (DMA) and hypersaline DSMZ 764 medium (salinity, 320) were used for the isolation of archaeal and bacterial members of the Thetis consortium, respectively. Cultures were serially diluted to 10^−8^ and the Hungate roll tube method used for cultivation of strict anaerobes [26] was applied to obtain single colonies. The roll tubes were repeated at least twice before the cultures were considered pure.

### 2.2. CARD-FISH Analysis

Samples were fixed with particle-free formaldehyde (37% w/v; Sigma Aldrich, Milano, Italy) solution (final concentration 2% v/v) for 1 h at room temperature and cells were permeabilized with lysozyme (10 mg·mL^−1^, 1 h) and achromopeptidase (5 mg·mL^−1^, 30 min) at 37 °C. Intracellular peroxidase was inhibited by treatment with 10 mM HCl at room temperature for 20 min. Filters were cut in sections and cells were hybridized by (HRP)-labeled oligonucleotide probes for *Eubacteria* (EUB338 I, II, III probe mix) and *Archaea* (Arch915) [27,28]. For signal amplification tyramide-Alexa488 [29] was used. The filter sections were counter-stained with DAPI (2 µg·mL^−1^) in a four to one ratio Citifluor (Citifluor Ltd., Leicester, UK): Vectashield (Linaris GmbH, Wertheim-Bettingen, Germany) [30]. At least 200 DAPI-stained and Alexa positive cells were counted in a minimum of 10 fields under an AXIOPLAN 2 Imaging microscope (Carl Zeiss, Jena, Germany). Negative control counts were performed with HRP-Non338 probe, always amounting to < 1% of DAPI-stained cells.

### 2.3. Chemical Analysis of the Metabolites of GB Degradation

The presence of H_2_ and CH_4_ was detected with a Chrompack CP 9001 gas chromatograph equipped with a thermal conductivity detector (135 °C) and a semi-capillary column Poraplot Q (25 m long, 0.53 mm internal diameter) operated at 35 °C with nitrogen (12 mL·min^−1^) or helium (12 mL·min^−1^) as the carrier. Methylamines were determined by gas chromatography using the same gas chromatograph equipped with a 3 m × 3.1 mm column (Chromosorb 103, 80/100 mesh) and with a flame-ionization detector (200 °C). The column was operated at 110 °C with helium (25 mL·min^−1^) as the carrier gas. GB and serine were analyzed with a HPLC apparatus, equipped with a Shimadzu LC 6A pump, a PYE Unicam UV detector (at 210 nm) fitted with a Hypersil-NH_2_ column (Touzart and Matignon); 10 mM phosphate:acetonitrile buffer (25:75; pH 7.0) was used as the solvent at a flow rate of 1 mL·min^−1^. Detection of acetate was carried out using a Dionex apparatus with the IonPac ASI 9 column equipped with AGI 9 guard column. The injection volume was 25 µL, and the flow rate was 1.0 mL·min^−1^. The mobile phase consisted of a linear gradient of KOH in H2O as follows: 10 mM (0–10 min), 10–58 mM (10–40 min) and 58–100 mM (40–60 min). The calibration curves for acetic acid showed good linearity (correlation coefficients ≥ 0.97) in the concentration range of 0.02–20 mM·L^−1^.

### 2.4. DNA Manipulation and Phylogenetic Analysis of 16S rRNA Genes

10 mL of Thetis enrichment were filtered through 0.2 µm polycarbonate filters (Merck Millipore, Darmstadt, Germany) for DNA purification. Filters were placed in a 2.0 mL tube covered with 350 µL of TENS extraction buffer (100 mM Tris-HCl pH 8.0, 40 mM EDTA pH 8.0, 200 mM NaCl, 2% SDS). DNA isolation was performed according to modified extraction methods from Urakawa *et al.* [31]. 350 µL phenol:chloroform:isoamyl alcohol (50:49:1) (saturated with TE buffer, pH 8.0) were added to the tube, the mixture was transferred to a bead-beating tube (lysing matrix E tube; MP Biomedicals, Illkirch, France) and mechanical agitated at the max velocity for 5 s using a Tissue Lyser (Qiagen, Hilden, Germany). After mechanical agitation, the tube was centrifuged at 13,000 rpm for 8 min, the aqueous phase was transferred in a 2.0 mL heavy Phase Lock Gel tube (5 Prime) containing 300 µL of 7.5 M ammonium acetate and mixed by repeated gentle inversion; an equal volume of chloroform was added. The mixture was mixed again by repeated gentle inversion and centrifuged at 13,000 rpm for 8 min. The upper phase was transferred in a 2 mL fresh tube containing 0.6 volume of ice cold isopropanol and precipitated at −20 °C overnight. Samples were centrifuged at 14,000 rpm for 20 min; the pellet was washed with 80% ethanol and centrifuged at 14,000 rpm for 10 min, air-dried and re-suspended in DNase-RNase and proteinase free water. Bacterial and archaeal 16S rRNA gene from DNA enrichment were amplified by PCR using corresponding universal primers: E8F (5′-AGAGTTTGATCATGGCTCAG-3′) and U1492R (5′-GTTACCTTGTTACGACTT-3′) for bacteria, and the 16S primer A8F (5′-CGGTTGATCCTGCCGGA-3′) and the reverse primer A1492R (5′-GGCTACCTTGTTACGACTT-3′) for archaea [5]. The PCR reaction and cloning was carried out as described elsewhere [5,32]. Ninety-six positive clones from each library were randomly selected by PCR amplification. A colony PCR from bacterial- and archaeal-pure culture isolates was also performed. The PCR products were further purified and sequenced at Macrogen (Seoul, South Korea). Initial alignment of amplified sequences and close relatives identified with BLAST [33] were carried out using the SILVA alignment tool [34] and manually aligned with ARB [35]. After alignment, the neighbor-joining algorithm of ARB and MEGA 5 [36] program packages were used to generate the phylogenetic trees based on distance analysis for 16S rRNA genes. The robustness of inferred topologies was tested by bootstrap re-sampling using the same distance model (1000 replicates).

### 2.5. Physiological Studies and High Pressure Cultivation

Growth tests for optimum salinity, pH, temperature, and pressure were performed with bacterial isolates in modified DSMZ 764 medium (10 g·L^−1^ glucose, 0.5 g·L^−1^ tryptone, 1 g·L^−1^ yeast extract, 1 mL·L^−1^ trace elements, 10 mL·L^−1^ vitamin solution, and 500 mg·L^−1^ Na_2_S) in completely filled 25 mL screw-cap tubes. The growth was monitored over a period of 10 days by spectrophotometer measurements and DAPI staining. Growth tests for utilizable substrates were performed in basal DSMZ 764 medium separately supplemented with xylane, sucrose, maltose, glycerol, gelatin, starch, cellulose, chitosan, chitin, pectin, inulin, cyclodestrine, ribose, and pyruvate (50 mM final concentration). A basal DSMZ 764 medium without substrate was used as negative control. The growth of bacterial isolates at different hydrostatic pressures (50, 100, 200, and 350 bars) was tested in high-pressure-maintaining titanium incubators [37]. One incubator was held at atmospheric pressure. The experiment was performed in DSMZ 764 medium during incubation for seven days at 37 °C, at optimal salinities. After the incubation, samples were gently depressurized (10 bars·min^−1^) and the growth was monitored by spectrophotometer measurements and DAPI staining.

### 2.6. Accession Numbers

The nucleotide sequences reported in this paper have been deposited in the DDBJ/EMBL/GenBank databases under accession numbers KJ677976–KJ677986.

## 3. Results and Discussion

### 3.1. Glycine Betaine-Degrading Enrichment from the Brine of Deep-Sea Lake Thetis

The evolution of methane in Mediterranean DHALs is well documented [7,8] but until recently [9] was not examined as a result of reductive degradation of GB followed by TMA fermentation. To obtain experimental evidence that this osmoprotectant could support the methanogenic microbial activity in other Mediterranean DHALs, we applied the previously developed enrichment approach [9] to the brine sample of Lake Thetis. As was described in the Material and Methods section, serial anoxic enrichment cultures, supplemented with 10 mM of GB, started from salinity values 300 and were stepwisely diluted to a salinity of 200. After two months of incubation, the evolution of methane was checked. The abundance of bacteria and archaea in the enrichments was monitored by CARD-FISH. The increase of salinity affected the contribution of culturable methanogens in GB enrichment cultures and no active methanogens were obtained with salinities exceeding 240. This finding neatly corroborates with the upper limit of *in vitro* salinity reported for growth of all known halophilic methanogenic species isolated from hypersaline sediments [15,18].

To the best of our knowledge, there are very few studies describing the GB-based trophic network established in highly-reduced salt-saturated environments [15,18,22,23,24]. Although this osmoprotectant is frequently present in hypersaline anoxic ecosystems, there are neither sulfate reducers nor methanogens known able to utilize GB at salinities exceeding 200 [22]. Implication of GB in the carbon cycle under salt saturation requires the activity of halophilic fermenters. These organisms can reductively degrade GB to trimethylamine and acetate by using either molecular hydrogen or amino acids (via Stickland reaction) as electron donors [15,21,22,38]. In the present study, the saltiest CH_4_-producing Thetis enrichment (salinity, 240) was further used for molecular analyses, isolation, and physiological studies. This enrichment was afterwards transferred into fresh medium of the same salinity, and supplemented with 10 mM of GB. The analysis of metabolites was done after one month of incubation at *in situ* temperature, as described previously [9]. Acetate (9.77 ± 0.15 mM), methane (1.62 ± 0.08 mM), and the minor traces of dimethyl- and monomethylamine (114 ± 23 µM and 37 ± 5 µM, respectively) were the only detectable products.

**Figure 1:**
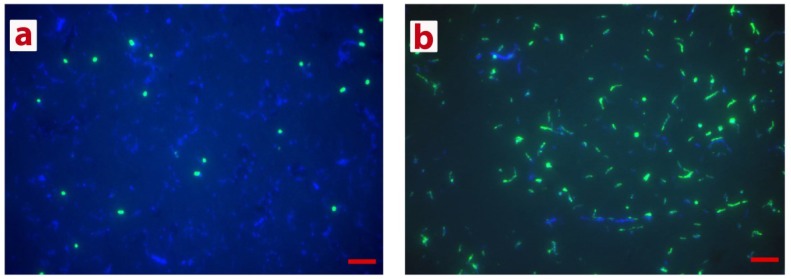
Direct microscopic observations of CARD-FISH- and DAPI-stained cells in Thetis GB-degrading enrichment. Coccoid cells positive for Archaea-specific probe Arch915 (**a**); and polymorphic single cells and chains of rod-shaped cells positive for EUB338 I, II, III probe mix (**b**) were visualized in GB-degrading Lake Thetis enrichment from the depth of 3500 m. Red scale bars denote: 5 µm.

**Figure 2:**
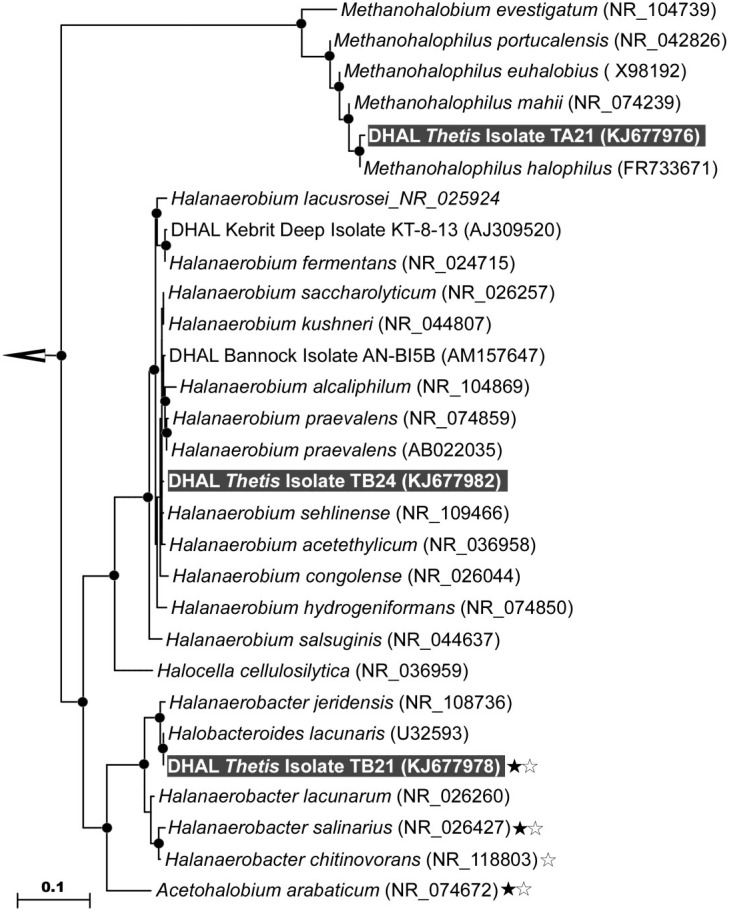
Phylogenetic affiliation of clones and isolates of Thetis GB-degrading enrichment. Neighbor-joining analysis using 1000 bootstrap replicates was used to infer tree topology. The scale bar represents 10% of sequence divergence. Bootstrap values (>75%) are indicated at branch points as closed circles. Sequences obtained in this study are evidenced by gray background. Sequences of organisms capable of reductive degradation of GB with either hydrogen or serine (Stickland reaction) are indicated by black and white stars, respectively.

Consistently with CARD-FISH analysis (Figure 1), the phylogenetic survey of cloned 16S rRNA genes showed that the GB-degrading microbial community comprised of both archaea and bacteria. Analysis of corresponding clone libraries revealed that archaeal fraction of the GB enrichment is represented entirely by methylotrophic methanogens of genus *Methanohalophilus.* All 96 clones were identical (KJ677977) and attributed to the most halophilic species *M. halophilus* and *M. mahii* of this genus. The bacterial fraction of the GB enrichment also possessed extremely low diversity and was represented exclusively by the organisms belonging to the order Halanaerobiales (Figure 2). One group of bacterial clones (84% of all 96 bacterial clones analyzed), represented by three sequences, KJ677979-81, and formed a tight cluster with *Halobacteroides lacunaris* Z-7888 (U32593). Although the classification of *Halobacteroides lacunaris* was reevaluated in 1995 [39,40], there are still some uncertainties remaining concerning the occurrence of two pairs of identical 16S rRNA gene sequences attributed to *Halanaerobacter lacunarum* DSM 6640^T^ (NR_026260/X89075) and *Halobacteroides lacunaris* Z-7888 (U32593/L37421). These sequences have only 94.5% identity between the pairs and likely belong to different species. The remaining clones of the bacterial fraction of the GB enrichment, represented by four almost-identical 16S rRNA gene sequences, KJ677983-86, were placed within the genus *Halanaerobium* with no categorical attribution to any of known species. We were aware that clone library sequencing cannot resolve the rare populations, thus, an occurrence of other organisms present in the GB enrichment in minor quantities is not excluded.

According to our precedent culture-independent clone library and metagenomic analyses [5,41], the prokaryotic community thriving in the Thetis brine is significantly enriched by bacterial KB1 and archaeal MSBL1 candidate divisions. We failed to find there any signatures of *Halanaerobiales*-related organisms. The 16S rRNA gene sequences of *Methanohalophilus*-related methanogens were also not detected, although these organisms were visualized in the Thetis brine by phylogenetic analysis of recovered *mcrA* transcripts [5]. All these findings indicated that *Halanaerobiales* and *Methanohalophilus* likely present in the Thetis brine as rare, but active, prokaryotic taxa. The metabolism of KB1- and MSBL1-related organisms is a subject of current debate, but the successful enrichment convincingly demonstrated their ability to grow syntrophically on GB [9]. Apparently, under extreme salinities the dominant groups KB1 and MSBL1 outcompete other organisms with similar metabolic requirements such as *Halanaerobiales* and *Methanohalophilus*. Members of the latest groups likely turn out to be of ecological relevance in the environments with decreased salinity, such as brine-seawater interfaces or less salted DHAL brines. Indeed, the 16S rRNA gene sequences of *Methanohalophilus* and *Halanaerobiales* were retrieved from the DHAL Urania (salinity < 190) and from the interfaces of DHALs Discovery and Kryos [6,42,43]. Additionally, these organisms were previously enriched from the diluted brines of Bannock and Thetis [5,7].

### 3.2. Isolation of Methanogenic Member of the GB-Degrading Consortium

**Figure 3:**
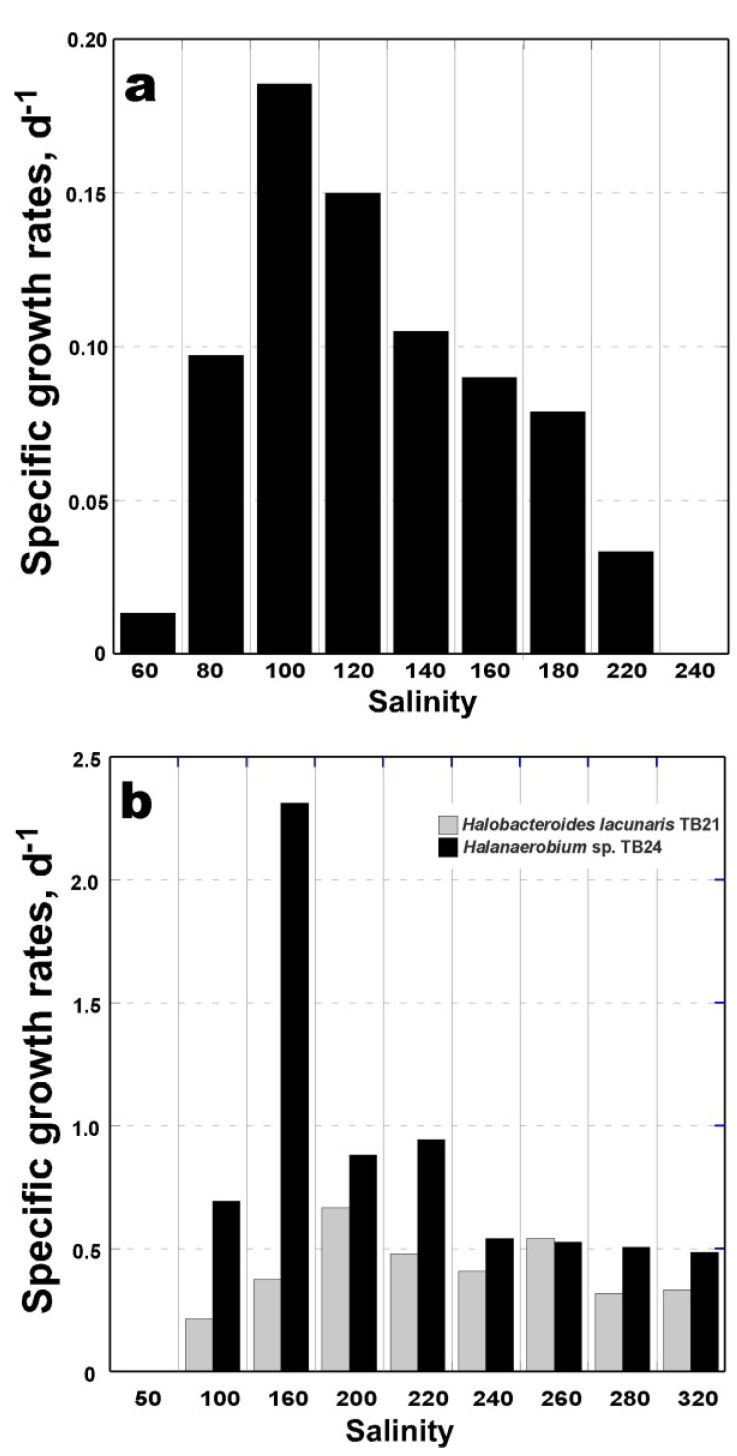
Growth of *Methanohalophilus* sp. TA21 (**a**) in DSMZ-280 medium with 20 mM TMA; *Halobacteroides lacunaris* TB21 and *Halanaerobium* sp. TB24 (**b**) in DSMZ-764 medium with 10 g·L^−1^ glucose as a function of salinity. The growth rates have been calculated from exponential growth measured by OD_600_. Each histogram represented average across two replicates, which were within 4%–15% of the reported values.

All known obligate halophilic bacterial fermenters, capable of trimethylamine degradation, can metabolize neither dimethylamine (DMA) nor monomethylamine (MMA) [24]. Taking this fact into account, a pure culture of *Methanohalophilus* sp. TA21 was obtained in minimal medium supplemented with DMA (20 mM final concentration). Currently cultivated methylotrophic methanogens of genus *Methanohaliphilus* are moderately halophilic and grow optimally at salinities of 40–120 [44,45]. Accordingly, the optimum growth of *Methanohalophilus* sp. TA21 in DSMZ-280 medium was observed at salinities of 100–120, no growth was observed at salinities < 60, and substantial growth was detected up to salinity 220 (Figure 3a). No growth of TA21 was observed on GB, indicating that without its bacterial counterpart(s), *Methanohalophilus* sp. TA21 cannot obtain energy and carbon from this osmoprotectant (Table S1). The TA21 cells produced methane as a major catabolic product while grown on TMA, DMA, MMA, and methanol (all substrates were applied at 20 mM concentrations). Compared to growth on TMA, methanol-feeding cells grew only about one-third as rapidly (data not shown).

### 3.3. Isolation of Fermenting Members of the GB-Degrading Consortium

As we mentioned above, the salinities of 240 are the upper limit reported for growth of all known cultivated *Methanohalophilus* strains. Accordingly, the *Halanaerobiales*-related organisms were obtained by cultivation of GB-degrading enrichment in rich DSMZ-764 medium under salt saturation conditions (salinity 320). After appropriate dilutions (10^−7^ and 10^−8^), the colonies with different morphologies were obtained by repeated use of the Hungate roll tube method [26]. Single colonies were picked, and serial dilutions in roll tubes were repeated at least twice before the cultures were considered pure. They were further phylogenetically characterized by sequencing of 16S rRNA genes and two isolates *Halobacteroides lacunaris* TB21 and *Halanaerobium* sp. TB24 were retained for further characterization. Remarkably, the 16S rRNA gene sequence of strain TB24 exhibited 98.81% similarity to *Halanaerobium* sp. strain AN-BI5B, previously isolated from the brine of Mediterranean DHAL Bannock [7].

### 3.4. Physiological Characterization of Bacterial Isolates TB21 and TB24

Despite belonging to the same order, both isolates TB21 and TB24 possess remarkable physiological and metabolic distinctions. As it is well established, members of order *Halanaerobiales* are able to ferment carbohydrates, but rarely can utilize either TMA or GB [15,24,36]. In accordance with this observation, we noticed that *Halanaerobium* sp. TB24 possessed a strictly fermentative metabolism and failed to grow on TMA and GB (Table S1). In contrast, an accumulation of 7.95 ± 0.52 mM of acetate at salinities of 150–300 was observed in *Halobacteroides lacunaris* TB21 culture supplemented with 20mM TMA. This finding has attributed the strain TB21 to the scarcely-studied group of methylotrophic halophilic acetogens [42]. In addition to TMA, *Halobacteroides lacunaris* TB21 was not able to use any of other tested C_1_-substrates (MMA, DMA, methanol, and formate), which is coherent with the metabolic features of the first described halophilic methylo- and chemolithotrophic homoacetogen *Acetohalobium arabaticum* [24]. Thus, TB21 and TB24 isolates cannot grow chemolithoautotrophically using H_2_ and CO_2_ but, unlike *Acetohalobium arabaticum*, both are capable of carbohydrates utilization. Cellobiose, fructose, glucose, maltose, mannitol, sucrose, and trehalose supported the growth of TB21 and TB24 strains. The major fermentation products from glucose produced by both strains were acetate, ethanol, CO_2_, and H_2_ (Table S1). The strains TB21 and TB24 are obligate halophiles with optimal NaCl requirements of 2.75–3.75 M (corresponding to the total salinity of 160–220) and with growth occurring in the presence of NaCl concentrations between 1.7 and 5.5 M (total salinities 100–320) (Figure 3b). The maximal growth rates for strains TB21 and TB24 in DSMZ-764 medium under optimal NaCl conditions at 37 °C and pH of 7.2 were 0.67 and 2.31 day^−1^, respectively. Such elevated optimal NaCl requirements placed TB21 and TB24 isolates among the most halophilic members of the order *Halanaerobiales*.

**Figure 4:**
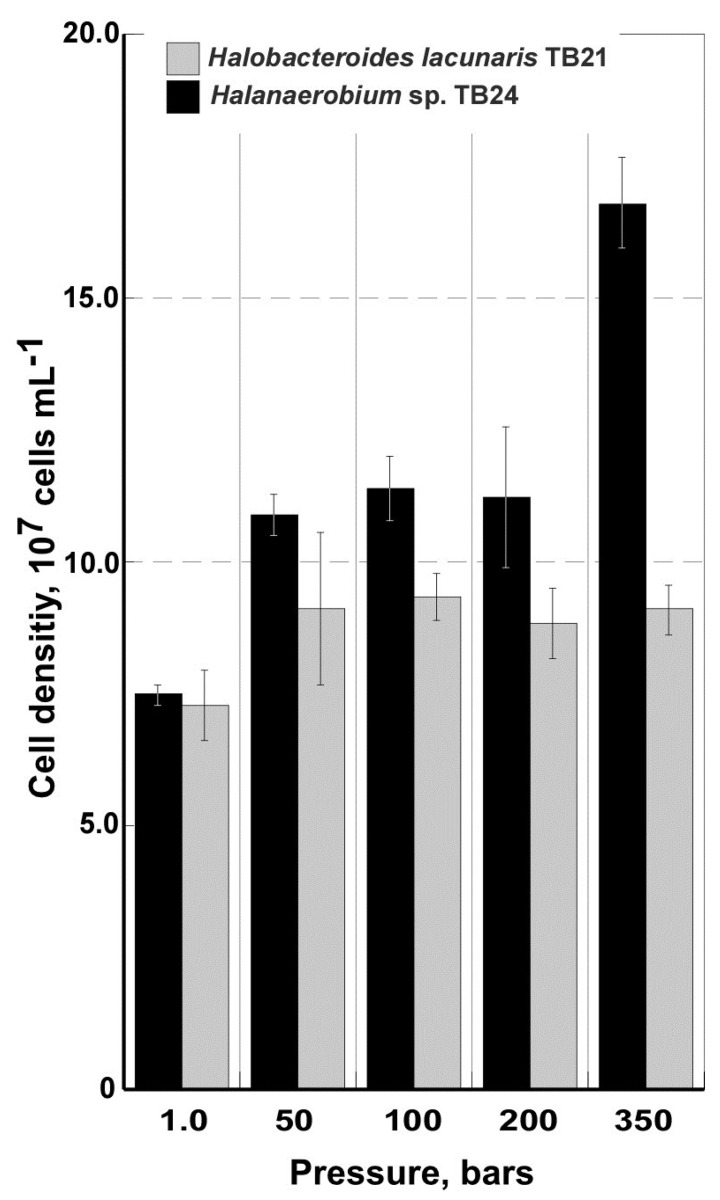
Growth of *Halobacteroides lacunaris* TB21 and *Halanaerobium* sp. TB24 in DSMZ-764 medium with 10 g·L^−1^ glucose as a function of pressure. The growth rates have been calculated from direct DAPI-counting after 72 h of exposition to the various pressures. Error bars indicate the standard deviation from triplicate samples.

Since both isolates were obtained from brine samples collected at the depth of 3500 m, the effect of hydrostatic pressure on their growth and survival was also estimated. The pressure response of each strain was measured over range from 1.0 to 350 bars, corresponding to atmospheric and *in situ* pressures. As it shown in Figure 4, *Halobacteroides lacunaris* TB21 exhibited broad piezotolerance with no statistically significant variations of growth rates within the whole range of applied pressure values (*p* ≥ 0.071, *n* = 3). In contrast, *Halanaerobium* sp. TB24 possessed an evident stimulation of the growth (all *p*-values are < 0.05) while culturing under *in situ* pressure conditions, *i.e.*, 350 bars. As we mentioned above, to withstand against osmotic stress in the hypersaline environment, all known members of the order *Halanaerobiales* use the “salt inside” strategy and accumulate elevated amount of K^+^ and Na^+^ inside the cells. High salt concentration within the cells reduces the intracellular water activity and this effect is able to stabilize proteins under high-pressure conditions [46]. Hence, a high amount of intracellular salts may explain the observed piezotolerance and piezophily of TB21 and TB24. However, as the high-pressure adaptation is likely the result of a combination of factors; these findings do not exclude the roles of other factors.

### 3.5. Reduction of GB with Hydrogen by Halobacteroides Lacunaris TB21

**Figure 5:**
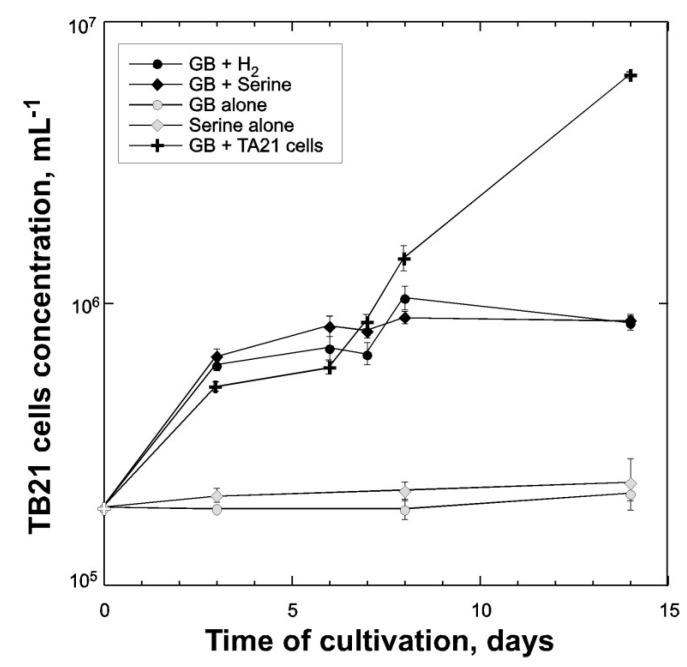
Growth of *Halobacteroides lacunaris* TB21 in liquid cultures with different substrates and with *Methanohalophilus* sp. TA21 cells. The growth of *Halobacteroides lacunaris* TB21 has been calculated from CARD-FISH-counting. Error bars indicate the standard deviation from triplicate samples.

GB is one of the major compatible solutes synthesized in a large variety of marine and halophilic organisms [22]. Thence, GB is of ecological importance in hypersaline environments, because many aerobic halophiles are able to use this compound as a source of carbon. However, degradation of GB under anaerobic conditions is problematic at high salt concentrations, because it requires either hydrogen or amino acids as electron donors. Only three species of extremely halophilic fermentative bacteria are currently considered to be able to degrade GB and use it as sole substrate for growth: *Acetohalobium arabaticum*, *Halanaerobacter salinarius*, and *Halanaerobacter chitinovorans* [24,38,47]. Similarly to these organisms, the strain TB21 was able to use GB in the presence of hydrogen as an electron donor. Without hydrogen, the growth and reductive degradation of GB was not observed (Figure 5). In the presence of serine, the strain was also able to degrade GB via a Stickland reaction. Other amino acids tested (alanine, glycine, histidine, leucine, ornithine, phenylalanine, proline, and valine) were not able to act as electron donors for reduction of GB. When GB and hydrogen were used as substrates, acetate, trimethylamine, CO_2_, and dimethylamine were detected in cell-free culture supernatant. Presence of latest product in the TB21 culture confirmed that, similarly to *Acetohalobium arabaticum*, this organism is capable to use TMA as a donor of the methyl group. Monomethylamine was never detected as a fermentation product. To confirm the existence of syntrophic interaction between methylotrophic, methanogenic, and fermenting acetogenic members of the Thetis enrichment, we mixed *Methanohalophilus* sp. TA21 and *Halobacteroides lacunaris* TB21 cells and grown them on minimal medium supplemented with 10 mM of GB. As it shown on Figure 5, during the first week of cultivation, the growth of TB21 in the presence of TA21 was comparable with growth on GB in the presence of added electron donors. Chemical analysis of metabolites detected the presence of monomethylamine (376 ± 26 µM) in the supernatant of grown TB21 + TA21 consortium, which was never observed in a pure culture of TB21.

## 4. Conclusions

Microbial communities in the deep-sea salt-saturated anoxic lakes of the Eastern Mediterranean are operating under harsh physical-chemical conditions that are incompatible with the life of common marine microorganisms. Nevertheless, a very specific microbial network occurs under given rigid constraints [9]. At least partially, this network may rely on fermentative decomposition of common osmoprotectant glycine betaine, produced in above layers of DHALs by moderate halophiles. Similar to other two extreme halophilic GB-degrading enrichments known so far [9,24], the initial step of GB degradation in the Thetis enrichment starts with its reductive cleavage to trimethylamine and acetate, carried out by the fermenting member *Halobacteroides lacunaris* TB21. In contrast to acetate, trimethylamine represents an advantageous C_1_-substrate for methylotrophic methanogenic member *Methanohalophilus* sp. TA21. This second member of GB-degrading halophilic consortium likely produces hydrogen via methylotrophic modification of reductive acetyl-CoA pathway because the initial GB cleavage reaction requires consumption of reducing equivalents. The ecophysiological role of the third member of the Thetis consortium remains yet to be elucidated, although its net opportunistic behavior in taking advantage of TA21:TB21 interspecific interaction is not excluded. Strain TB24 lacks the capability of degradation of either GB or trimethylamine, but as it is true for the majority of cultivated members of *Halanaerobiales*, it can obtain energy primarily by fermenting simple sugars and producing hydrogen as one of the end metabolites. Hence, while consuming of TB21 and TA21 metabolites, *Halanaerobium* sp. TB24 can likely serve as an additional provider of reducing equivalents required for reductive degradation of GB by the TB21 cells.

## Supplementary Files

Supplementary File 1
