# Supplementary material for: A Three-Component Microbial Consortium from Deep-Sea Salt-Saturated Anoxic Lake Thetis Links Anaerobic Glycine Betaine Degradation with Methanogenesis
Source: Microorganisms. 2015 Sep 9;3(3):500–17. doi: 10.3390/microorganisms3030500 (PMC5023251; doi:10.3390/microorganisms3030500)
Supplement: Supplementary File 1 [file microorganisms-03-00500-s001.docx]

**Supplementary Material**

**Table S1.** General characteristics of three isolated members of GB-degrading extremely halophilic anaerobic consortium obtained from the brine of DHAL *Thetis*.

| **Characteristics** | ***Methanohalophilus* sp. TA21** | ***Halobacteroides lacunaris* TB21** | ***Halanaerobium* sp. TB24** |
| --- | --- | --- | --- |
| Salintiy range | 60–220 | 100–320 | 100–320 |
| Optimum salinity | 100–120 | 160–220 | 160–220 |
| Doubling time, day^−1^ | 0.18 | 0.67 | 2.31 |
| Pressure range, bars | ND | 1–350 | 1–350 |
| Optimum pressure | ND | 1–350 | 350 |
| Use of substrates |  |  |  |
| Glycine betaine (GB) | No growth | No growth | No growth |
| GB + H_2_ | No growth | Yes | No growth |
| GB + serine | No growth | Yes | No growth |
| Trimethylamine (TMA) | Yes | Yes | No growth |
| Dimethylamine (DMA) | Yes | No growth | No growth |
| Monomethylamine (MMA) | Yes | No growth | No growth |
| Methanol | Yes | No growth | No growth |
| Metabolites from TMA | DMA, MMA, NH_4_^+^ | DMA, acetate | No growth |
| Fermentation products from glucose | No growth | Acetate, ethanol, H_2_, CO_2_ | Acetate, ethanol, H_2_, CO_2_ |

ND, not determined.
